# Characterization data of reference industrial polycarboxylate superplasticizer VP 2020/15.2 used for Priority Program DFG SPP 2005 “Opus Fluidum Futurum - Rheology of reactive, multiscale, multiphase construction materials”

**DOI:** 10.1016/j.dib.2021.107657

**Published:** 2021-11-30

**Authors:** Lin Zhang, Ran Li, Lei Lei, Johann Plank

**Affiliations:** Technische Universität München, Chair for Construction Chemistry, 85747 Garching, Lichtenbergstraße 4, Germany

**Keywords:** Polycarboxylate, Characterization, Molecular properties, Dispersing effectiveness, Fluidity, DFG SPP 2005

## Abstract

An industrial polycarboxylate superplasticizer sample has been chosen in the Priority Program 2005 of the German Research Foundation (DFG SPP 2005). The molecular properties of this superplasticizer sample, such as molecular weight (*M_w_, M_n_*), the polydispersity index (PDI) and the macromononmer conversion rate were determined using Size Exclusion Chromatography (SEC), and the sample's specific anionic charge amount was obtained via charge titration. Furthermore, the dispersing effectiveness of this superplasticizer sample was assessed through ‘mini-slump’ tests in pure OPC (CEM I 42.5 R) and in a limestone-calcined clay (LCC) cement. Moreover, the adsorption of the superplasticizer on both cements, and the dosage-dependent development of the zeta potential of both cement suspensions were captured. The data shall be used for the ongoing research within the Priority Program.

## Specifications Table

**Table:** 

Subject	Polymers and Plastics
Specific subject area	Admixture for concrete; Dispersant; Polyethylene glycol derivatives
Type of data	Table; Image; Figure
How data were acquired	PCD 03 pH particle charge detector; Size exclusion chromatography (SEC); High TOC II Instrument; DT 1200 Electroacoustic Spectrometer; Infrared balance; pH meter; DIN EN 1015
Data format	Raw; Analyzed
Parameters for data collection	Molecular weight (*M_w_, M_n_*); Polydispersity index; Conversion; Solid content; Density; Specific anionic charge amount; Zeta potential; Adsorbed amount; Spread flow, Slump retention
Description of data collection	The data were obtained at the Chair for Construction Chemistry, Prof. Dr. J. Plank, Technische Universität München.
Data source location	Technische Universität München, Chair for Construction Chemistry, 85747 Garching, Lichtenbergstraße 4, Germany
Data accessibility	Repository name: mediaTUM Data identification number: https://doi.org/10.14459/2021mp1632413 Direct URL to data: https://mediatum.ub.tum.de/1632413

## Value of the Data

• The chemical and physical properties, as well as application performance, of a polycarboxylate (PCE) superplasticizer used in the Priority Program 2005 of the German Research Foundation (DFG SPP 2005) were characterized in detail and are recorded in this dataset.
• This data is available to all research partners within the DFG SPP 2005 Priority Program and other researchers who use the same material in their research.
• The research groups involved in the SPP 2005 employ the polymer as a slump retainer for cement suspensions, including the LCC binder, and other colloidal systems to produce rheological data.
• The structural parameters, such as molecular weights and anionic charge, of this polymer should be helpful for researchers to compare this superplasticizer with other superplasticizers to determine the optimal structures for their specific application.
• The data should help the researchers in the SPP project gain more insight into the interactions that occur between particles and the polycarboxylate polymer.

## 1 Data Description

A thorough characterization of two superplasticizers was published in [1]. The data presented here relate to a new superplasticizer, VP2020/15.2, provided by MBCC group (Mannheim / Germany); this superplasticizer was tested in a CEM I 42.5 R sample and LCC cement. Detailed information about the chemical composition, physical properties and molecular characteristics of this polycarboxylate superplasticizer are provided below. The dispersing effectiveness of this polycarboxylate polymer was evaluated using ‘mini-slump’ tests and its interaction with both cements was assessed by zeta potential and adsorption measurements.

### 1.1 Characterization data of physical and chemical properties

**Fig. 1:**
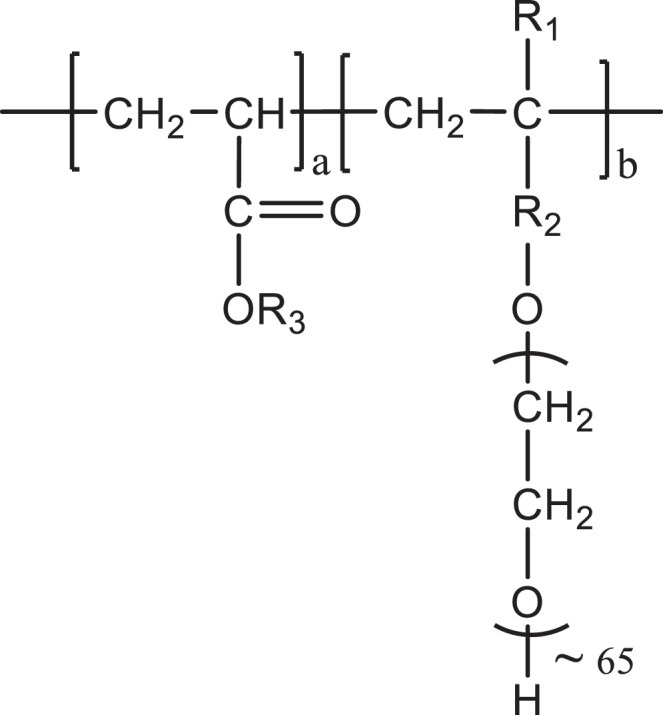
Chemical structure of the industrial PCE sample VP 2020/15.2.

**Fig. 2:**
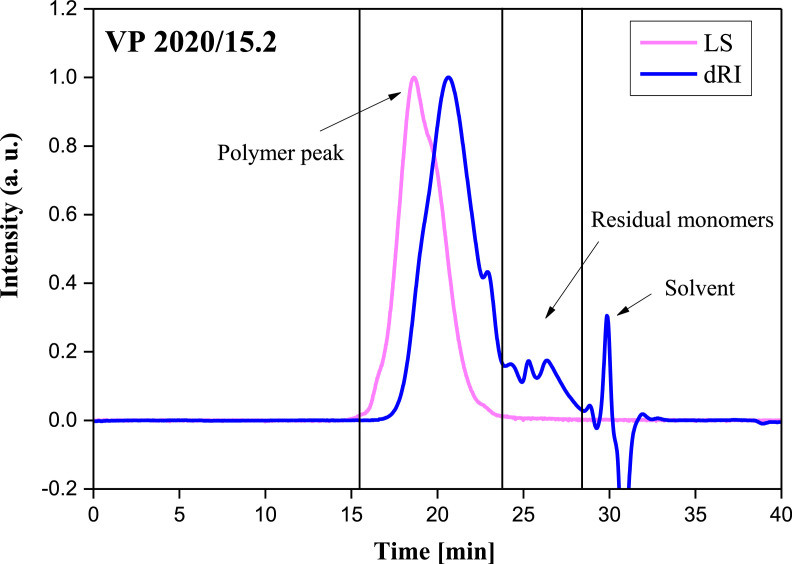
SEC spectrum of PCE sample VP 2020/15.2; eluent: 0.1 M NaNO_3_.

The physical properties, such as solid content, density, pH value, and chemical characteristics, including molecular weights (*M_w_, M_n_*), PDI, macromonomer conversion of this industrial PCE sample, are listed in Table 1. The chemical structure of this PCE is displayed in Fig. 1, and the SEC spectrum is presented in Fig. 2. VP 2020/15.2 is a polycarboxylate comb polymer, and the ethylene oxide unit number in the side chain is approximately 65.

**Table 1:** Solid content, density, molecular weights, polydispersity index (PDI), macromonomer conversion and pH value of the industrial PCE sample VP 2020/15.2.

Product	Solid content [wt.%]	Density [kg/L]	*M_w_* [g/mol]	*M_n_* [g/mol]	PDI	Macromonomer Conversion [%]	pH
VP 2020/15.2 (ready-mix type PCE)	20.5	1.01	78,100	28,560	2.73	86.1	5.6

### 1.2 Characterization data of anionic charge property

**Fig. 3:**
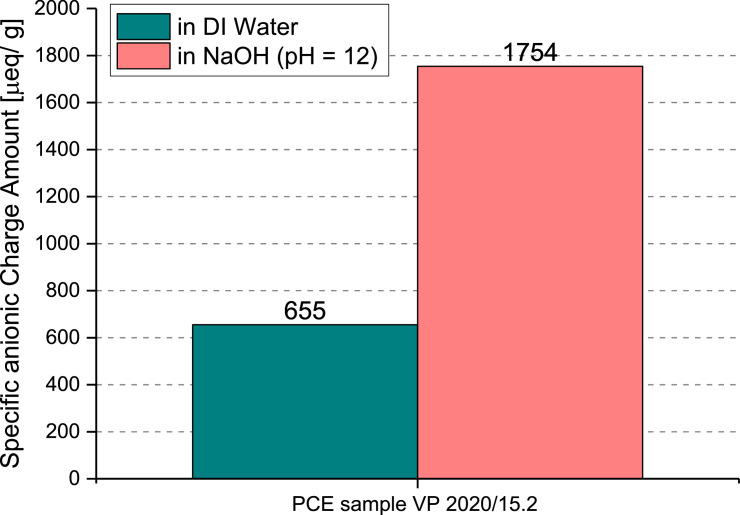
Specific anionic charge amount of VP 2020/15.2.

The PCD 03 pH particle charge detector (Mütek Analytic, Herrsching, Germany) was used to capture the specific anionic charge amount of this PCE. The PCE superplasticizer sample was dissolved in deionized water as well as in 0.01M NaOH solution (pH = 12) respectively. Polydiallyl dimethyl ammonium chloride (polyDADMAC) solution was employed to titrate PCE until the charge was neutralized. The results are shown in Table 2 and Fig. 3.

**Table 2:** Specific anionic charge amount of the industrial superplasticizer sample.

Product	Specific anionic charge amount [μeq/g]	
	in DI water	in 0.01 M NaOH, pH = 12
VP 2020/15.2 (ready-mix type PCE)	655	1,754

### 1.3 Dosage - dependent dispersing effect in cement pastes

The dispersing power as a function of dosage of this superplasticizer was investigated in cement paste via the ‘mini slump’ test at 20°C and 40% rel. humidity according to DIN EN 1015-3 [2]. The water-to-cement ratio was fixed at 0.4 for both the CEM I 42.5 R and the LCC cement, based on water demand [3,4].

**Fig. 4:**
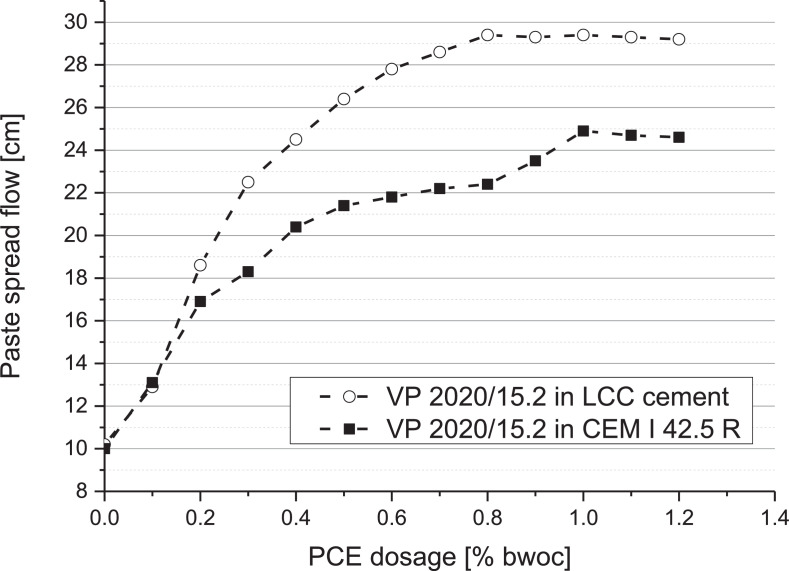
Dosage - dependent spread flow of VP 2020/15.2 in CEM I 42.5 R and LCC cement paste (w/c ratio = 0.4).

The dispersing effectiveness of this superplasticizer at different dosages in two cements (CEM I 42.5 R and LCC cement) was assessed, and the results are shown in Fig. 4. The dosage required for VP 2020/15.2 in CEM I 42.5 R to reach maximum fluidity was ∼ 1.0% bwoc, while in the LCC cement the dosage required to reach maximum effect was ∼ 0.8% bwoc (w/c ratio = 0.4).

### 1.4 Slump retention

**Fig. 5:**
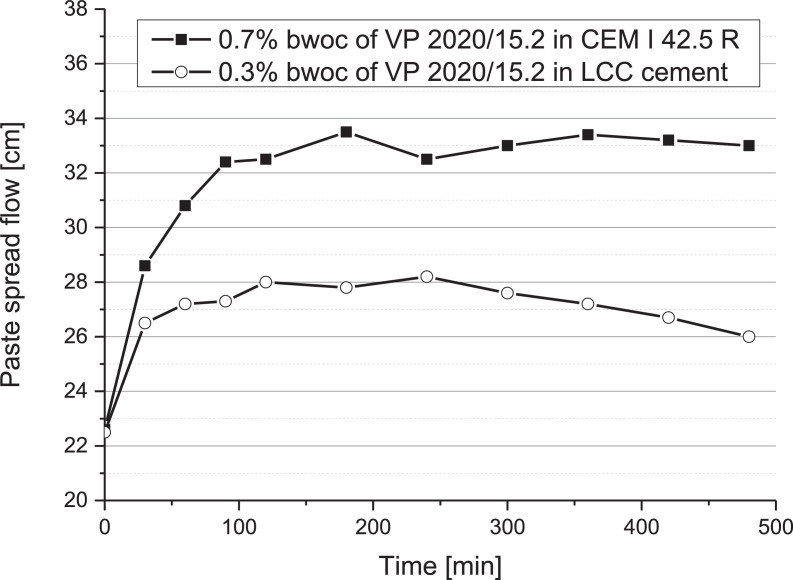
Slump retention of VP 2020/15.2 in CEM I 42.5 R and LCC cement paste (w/c ratio = 0.4).

The slump retention performance of the superplasticizer sample VP 2020/15.2 (ready-mix type PCE) in two cements (CEM I 42.5 R and LCC cement) was measured over a period of 6 hours. The w/c ratio was 0.4. The results are shown in Fig. 5.

The dosages of VP 2020/15.2 used in CEM I 42.5 R and LCC cement were 0.7% bwoc and 0.3% bwoc, respectively. In both cements, VP 2020/15.2 shows strong delayed plastification. This effect is weaker in the LCC cement.

### 1.5 Adsorption of the PCE sample on CEM I 42.5 R and LCC cement

The adsorbed amounts of VP 2020/15.2 on CEM I 42.5R and LCC cements were obtained via using the depletion method [5]. A Liquid TOC-II instrument (Elementar Analysen systeme GmbH, Hanau/ Germany) was employed to obtain the total organic carbon amount.

**Fig. 6:**
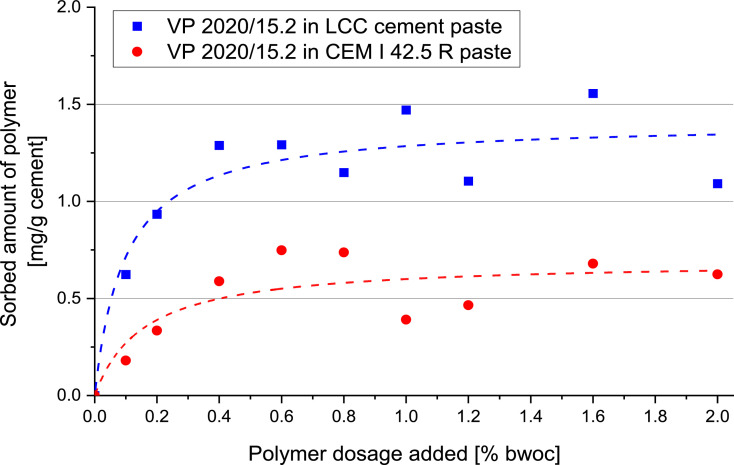
Adsorption amount for VP 2020/15.2 on CEM I 42.5 R and LCC cement; w/c ratio = 0.5, cement paste.

The adsorption isotherms for VP 2020/15.2 on CEM I 42.5 R and LCC cement are shown in Fig. 6.

### 1.6 Zeta potential of cement suspensions admixed with VP 2020/15.2

**Fig. 7:**
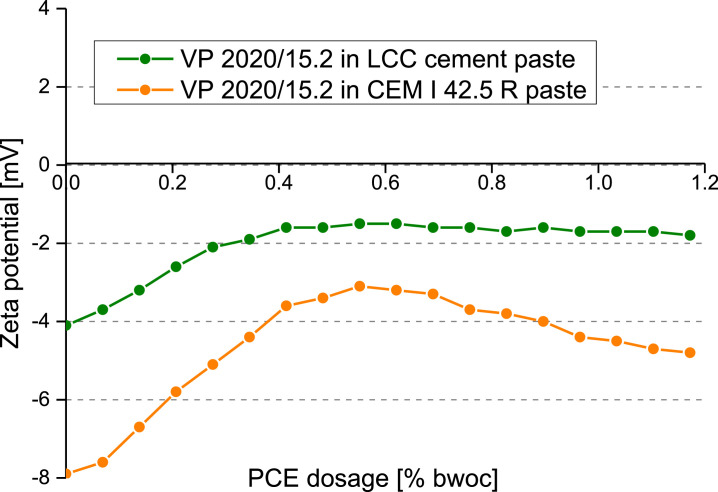
Dosage-dependent zeta potentials of pastes prepared from CEM I 42.5 R and LCC cement, treated with VP 2020/15.2 (w/c ratio = 0.5).

In order to further understand the interaction of the PCE and the cement particle surface, zeta potentials as a function of PCE dosage were characterized via a DT-310 instrument (Dispersion Technology Inc., Bedford Hills, NY/USA). The zeta potential value was calculated by means of the colloidal vibration current (CVI) [6]. The w/c ratio was fixed at 0.5 to achieve a slump flow value of 18 cm in both the CEM I 42.5 R and the LCC cement. The dosage dependent zeta potential values were recorded and are shown in Fig. 7.

## 2 Experimental Design, Materials and Methods

The molecular characteristics of VP 2020/15.2 were determined utilizing size exclusion chromatography (SEC) to obtain the conversion of the macromonomers and the molecular properties (*M_w_, M_n_* and PDI). The instrument employed was a Waters Alliance 2695 (Waters, Eschborn, Germany) with a “mini Dawn” detector (Wyatt Technology Corp., Santa Barbara, CA). Three Ultrahydrogel columns (Waters, Eschborn, Germany) were used to separate the polymer and 0.1 N NaNO_3_ at a flow rate of 1.0 mL*min^− 1^ was used as the mobile phase [7].

The specific anionic charge amount of the PCE sample was obtained using a PCD 03 pH particle charge detector (Mütek Analytic, Herrsching, Germany). For this measurement, the PCE polymer (0.1 g/L) was dissolved in DI water and 0.01 M NaOH solution. Polydiallyl dimethyl ammonium chloride (polyDADMAC, 0.34 g/L) was used to titrate the PCE until the charge was neutralized. The negative charge amount of polymer (per gram) was calculated based on the polyDADMAC consumption to reach the zero potential [8].

Based on the norm DIN EN 1015-3, the dispersion and slump retention performance of the PCE polymer were investigated utilizing the “mini-slump” test. The cement (300 g) was added into a porcelain cup with pre-mixed PCE solution and kept still for 1 min, then manually stirred for 2 min. The cement paste was immediately poured into the Vicat cone (40 mm × 70 mm × 80 mm), filled to the brim, and then placed on a glass plate, thereafter the cone was removed vertically. The resulting paste diameter indicates the cement paste flow value. The paste diameter was measured twice, perpendicular to each other, and the average was then calculated. With respect to the slump retention performance, the similar fluidity test was conducted after 30, 60, 90, 120, 180, 240, 300, 360, 420 and 480 minutes after the first measurement.

The total organic carbon (TOC) content was used to characterize the PCE adsorption amounts on the cement surface. In this experiment, cement (16 g) and deionized water (8.0 g), containing the pre-dissolved VP 2020/15.2, were mixed (w/c ratio = 0.5), and the mixture was then shaken for two minutes at 2400 rpm (VWR International, Darmstadt / Germany), afterwards centrifuged for 10 minutes at 8,500 rpm. The supernatant was collected and filtered (0.2 µm), adding 0.1 M HCl to prevent carbonation and to remove inorganic carbonates. The instrument used to quantify the organic carbon amount was Liquid TOC-II (Elementar Analysensysteme GmbH, Hanau, Germany). The organic carbon amount was measured twice and the adsorbed amount of PCE was then calculated based on the initial PCE concentration and TOC content in the supernatant.

Zeta potential: The cement suspensions’ electro - kinetic characteristics were determined with the DT 1200 instrument (Dispersion Technology, Inc, NY/ USA). In this experiment, cement (300 g) and DI water (150 g) were mixed in a porcelain cup and kept still for 1 min, then manually stirred for another 2 min. For the measurement, the paste was poured into a glass container, and the titrator, zeta potential electrode, pH meter, temperature probe were merged into the cement paste. During the test, the paste was continuously stirred at ambient temperature at 200 rpm. The ionic background was subtracted from the resulting zeta potential value to yield the values shown in Fig. 7.

## Ethics Statement

The work did not involve the use of human subjects, animal experiments, or data collected from social media platforms.

## CRediT Author Statement

**Lin Zhang**: Investigation, Methodology, Data curation, Validation, Writing - original draft; **Ran Li**: Investigation, Data curation; **Lei Lei**: Writing - Conceptualization, Reviewing and Editing; **Johann Plank**: Conceptualization, Resources, Supervision.

## Declaration of Competing Interest

The authors declare that they have no known competing financial interests or personal relationships which have or could be perceived to have influenced the work reported in this article.
